# Carboxyl-Terminal Receptor Domains Control the Differential Dephosphorylation of Somatostatin Receptors by Protein Phosphatase 1 Isoforms

**DOI:** 10.1371/journal.pone.0091526

**Published:** 2014-03-17

**Authors:** Andreas Lehmann, Andrea Kliewer, Jan Carlo Märtens, Falko Nagel, Stefan Schulz

**Affiliations:** Institute of Pharmacology and Toxicology, Jena University Hospital, Friedrich-Schiller-University, Jena, Germany; Institute of Molecular and Cell Biology, Biopolis, United States of America

## Abstract

We have recently identified protein phosphatase 1β (PP1β) as G protein-coupled receptor (GPCR) phosphatase for the sst_2_ somatostatin receptor using siRNA knockdown screening. By contrast, for the sst_5_ somatostatin receptor we identified protein phosphatase 1γ (PP1γ) as GPCR phosphatase using the same approach. We have also shown that sst_2_ and sst_5_ receptors differ substantially in the temporal dynamics of their dephosphorylation and trafficking patterns. Whereas dephosphorylation and recycling of the sst_2_ receptor requires extended time periods of ∼30 min, dephosphorylation and recycling of the sst_5_ receptor is completed in less than 10 min. Here, we examined which receptor domains determine the selection of phosphatases for receptor dephosphorylation. We found that generation of tail-swap mutants between sst_2_ and sst_5_ was required and sufficient to reverse the patterns of dephosphorylation and trafficking of these two receptors. In fact, siRNA knockdown confirmed that the sst_5_ receptor carrying the sst_2_ tail is predominantly dephosphorylated by PP1β, whereas the sst_2_ receptor carrying the sst_5_ tail is predominantly dephosphorylated by PP1γ. Thus, the GPCR phosphatase responsible for dephosphorylation of individual somatostatin receptor subtypes is primarily determined by their different carboxyl-terminal receptor domains. This phosphatase specificity has in turn profound consequences for the dephosphorylation dynamics and trafficking patterns of GPCRs.

## Introduction

The signaling output of G protein-coupled receptors (GPCRs) is desensitized by mechanisms involving phosphorylation, β-arrestin binding and internalization. GPCR signaling is resensitized by mechanisms involving dephosphorylation, but details about the phosphatases responsible are generally lacking. We and others have recently succeeded in identifying bona fide GPCR phosphatases for a number of receptors using a combined approach of phosphosite-specific antibodies and siRNA screening in HEK293 cells. First, we identified protein phosphatase 1β (PP1β) as GPCR phosphatase for the sst_2_ somatostatin receptor [1]. Second, we identified PP1γ as GPCR phosphatase for the μ-opioid receptor and the sst_5_ somatostatin receptor [2] [3]. Third, more recently Gehret and Hinkle identified PP1α as GPCR phosphatase for the thyrotropin-releasing hormone receptor [4]. All of the above observations were made in a similar cellular background. This suggests that a given GPCR may recruit its specific PP1 isoform for rapid dephosphorylation with remarkable selectivity. However, it is not known which GPCR domain directs the engagement of specific PP1 isoforms to the receptor.

Here, we have addressed this question using the closely-related sst_2_ and sst_5_ somatostatin receptors. The sst_2_ and sst_5_ receptors exhibit a high degree of homology in their transmembrane domains but exhibit divergent carboxyl-terminal tails. Both the sst_2_ and the sst_5_ receptor are pharmacological relevant targets for clinically-used drugs [5] [6] [7] [8] [9] but the two receptors exhibit strikingly different phosphorylation and trafficking patterns. The sst_2_ receptor is a prototypical class B receptor that is phosphorylated at a cluster of at least six carboxyl-terminal serine and threonine residues upon agonist exposure. The sst_2_ receptor than forms a stable complex with β-arrestin that co-internalize into the same endocytic vesicles. Consequently, the sst_2_ receptor recycles slowly [1] [10] [11]. By contrast, the sst_5_ receptor is a prototypical class A receptor in that its endocytosis is regulated by a single phosphorylation at T333. The sst_5_ receptor then forms relatively unstable ß-arrestin complexes that dissociate at or near the plasma membrane. The receptor internalizes without ß-arrestin and recycles rapidly [2] [12]. Here, we show that a tail-swap mutation of sst_2_ and sst_5_ receptors is required and sufficient to reverse the patterns of dephosphorylation and trafficking of these two receptors.

## Materials and Methods

### Reagents, plasmids and antibodies

SS-14 was obtained from Bachem (Weil am Rhein, Germany). DNA for HA-tagged human sst_2_ and sst_5_ receptor, 2-5- and 5-2-chimaera were generated via artificial gene synthesis and cloned into pcDNA3.1 by imaGenes (Berlin, Germany). The human HA-tagged sst_2_ receptor was obtained from UMR cDNA Resource Center (Rolla, MO). The phosphorylation-independent rabbit monoclonal anti-sst_2_ antibody {UMB-1} and anti-sst_5_ antibody {UMB-4} were obtained from Epitomics (Burlingame, CA). The phosphosite-specific sst_2A_ antibodies anti-pS341/pS343 {3157}, anti-pT353/pT354 {0521}, anti-pT356/pT359 {0522} and phosphosite-specific sst_5_ antibodies anti-pT333 {3567} as well as the rabbit polyclonal anti-HA antibodies were generated and extensively characterized as previously described [1] [2].

### Cell culture and transfection

Human embryonic kidney HEK293 cells were obtained from the German Resource Centre for Biological Material (DSMZ, Braunschweig, Germany). HEK293 cells were grown in DMEM supplemented with 10% fetal calf serum. Cells were transfected with plasmids using Lipofectamine 2000 according to the instructions of the manufacturer (Invitrogen, Carlsbad, CA). Stable transfectants were selected in the presence of 400 µg/ml G418. Stable cells were characterized using radioligand-binding assays, Western blot analysis, and immunocytochemistry as described previously. All receptors and chimeras tested were present at the cell surface, expressed similar amounts of receptor protein and had similar affinities for SS-14 as the wild-type receptors.

### Analysis of receptor internalization by confocal microscopy

Cells were grown on poly-L-lysine-coated coverslips overnight. After treatment with 1 µM SS-14 for 0, 15 or 30 min at 37°C, cells were fixed with 4% paraformaldehyde and 0.2% picric acid in phosphate buffer (pH 6.9) for 30 min at room temperature and washed several times. Specimens were permeabilized and then incubated with anti-sst_2A_ {UMB-1} or anti-sst5 antibody {UMB-4} antibodies followed by Alexa488-conjugated secondary antibodies. Specimens were mounted and examined using a Zeiss LSM510 META laser scanning confocal microscope.

### Quantification of receptor internalization by ELISA

Stably transfected HEK293 cells were seeded onto poly-L-lysine-treated 24-well plates. The next day, cells were preincubated with 1 µg/ml anti-HA antibody for 2 h at 4°C. After the appropriate treatment with SS-14 (1 µM) for 30 min at 37°C, cells were fixed and incubated with peroxidase-conjugated anti-rabbit antibody overnight. After washing, plates were developed with ABTS solution and analyzed at 405 nm using a microplate reader.

### Western blot analysis

Cells were plated onto 60-mm dishes and grown to 80% confluence. After treatment with SS-14, cells were lysed in detergent buffer (50 mM Tris-HCl, pH 7.4, 150 mM NaCl, 5 mM EDTA, 10 mM NaF, 10 mM disodium pyrophosphate, 1% Nonidet P-40, 0.5% sodium deoxycholate, 0.1% SDS, 0.2 mM phenylmethylsulfonyl fluoride, 10 µg/ml leupeptin, 1 µg/ml pepstatin A, 1 µg/ml aprotinin, and 10 µg/ml bacitracin). All phosphorylation and dephosphorylation assays were performed at both physiological temperature (37°C) and at room temperature (22°C) for the indicated time periods. Glycosylated proteins were partially enriched using wheat germ lectin-agarose beads as described. Proteins were eluted from the beads using SDS-sample buffer for 20 min at 65°C and then resolved on 8% SDS-polyacrylamide gels. After electroblotting, membranes were incubated with phosphosite-specific antibodies anti-pS341/pS343 {3157}, anti-pT353/pT354 {0521}, anti-pT356/pT359 {0522}, anti-pT333 {3567} at a concentration of 0.1 µg/ml followed by detection using enhanced chemiluminescence. Blots were subsequently stripped and reprobed with anti-sst_2A_ antibody {UMB-1} or anti-sst5 antibody {UMB-4} to confirm equal loading of the gels.

### β-Arrestin-EGFP mobilization assay

HEK293 cells were seeded onto 35-mm glass-bottom culture dishes (Mattek, Ashland, MA). The next day, cells were transiently cotransfected with 0.2 µg β-arrestin-2-EGFP and 2 µg human or chimeric somatostatin receptor or with a mixture of 0.2 µg β-arrestin-2-EGFP, 0.8 µg GRK2 and 1.2 µg human/chimeric sst2 receptor per dish containing 200,000 cells using TurboFect™ (Fermentas) according to the instructions of the manufacturer. After 24 h, cells were transferred onto a temperature-controlled microscope stage set at 37°C of a Zeiss LSM510 META laser scanning confocal microscope. Images were collected sequentially using single line excitation at 488 nm with 515–540-nm band pass emission filters. Saturating concentrations of SS-14 (1 µM) were applied directly into the culture medium immediately after the initial image was taken.

### Small Interfering RNA Silencing of Gene Expression

Chemically synthesized double-stranded siRNA duplexes (with 3′ dTdT overhangs) were purchased from Qiagen (Hilden, Germany) for the following targets: PP1α catalytic subunit (5′-AAGAGACGCTACAACATCAAA-3′), PP1β catalytic subunit (5′- ACGAGGATGTCGTCCAGGAA-3′ and 5′-GTTCGAGGCTTATGTATCA-3′), PP1γ catalytic subunit (5′-ACATCGACAGCATTATCCAA-3′ and 5′-AGAGGCAGTTGGTCACTCT-3′), and a nonsilencing RNA duplex (5′- GCTTAGGAGCATTAGTAAA-3′ or 5′-AAA CTC TAT CTG CAC GCT GAC-3′). HEK293 cells were transfected with 150 nM siRNA for single transfection or with 100 nM of each siRNA for double transfection using HiPerFect (Qiagen). Silencing was quantified by immunoblotting. All experiments showed protein levels reduced by ≥80%.

### Data Analysis

Data were analyzed using GraphPad Prism 4.0 software. Statistical analysis was carried out with Students *t*-test as well as with one-way or two-way ANOVA followed by the Bonferroni post-test. *p-Values* of <0.05 were considered statistically significant.

## Results

**Figure 1:**
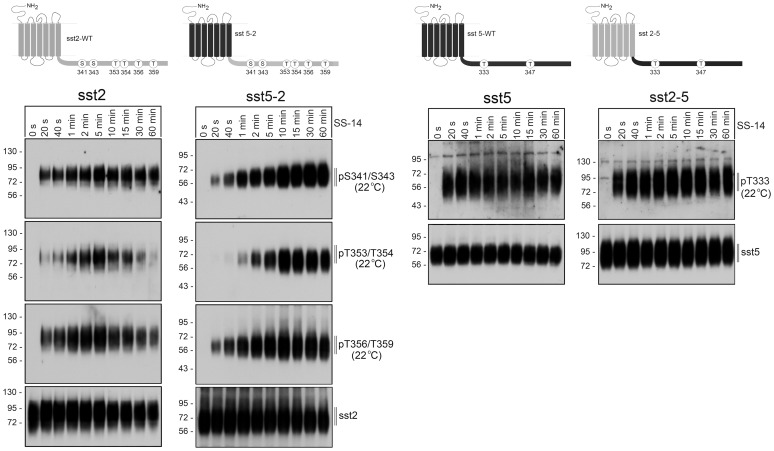
Agonist-induced phosphorylation of sst_2_ and sst_5_ tail-swap mutants. *Top*, Schematic representation of the human wild-type sst_2_ (depicted in grey) and human wild-type sst_5_ receptors (depicted in black) and their corresponding tail-swap mutants. Phosphate acceptor sites targeted for the generation of phosphosite-specific antibodies are depicted as circles. *Bottom*, stably transfected HEK 293 cells were exposed to 1 µM SS-14 at room temperature for the indicated time periods. Cells were lysed and immunoblotted with the indicated phosphosite-specific antibodies. Blots were then stripped and reprobed with the phosphorylation-independent anti-sst_5_ antibody {UMB-4} or anti-sst_2_ antibody {UMB-1} to confirm equal loading of the gels. Shown are representative results from one of three independent experiments. The position of molecular mass markers is indicated on the left (in kDa).

The sst_2_ and sst_5_ receptors exhibit a high degree of homology, yet these somatostatin receptors are dephosphorylated by different PP1 isoforms. To elucidate which receptor domains determine this remarkable phosphatase specificity, we first constructed tail-swap mutants of these two receptors. In initial studies, we confirmed that all receptors were expressed at similar levels on the cell surface, and exhibited similar binding properties. All four receptors also exhibited similar signaling properties determined as their ability to activate ERK in a pertussis toxin-sensitive manner (not shown). We then compared agonist-induced phosphorylation of the wild-type sst_2_ receptor with that of the sst_5-2_ receptor using phosphosite-specific antibodies for pS341/343, pT353/354 and pT356/359 (Figure 1, *left panel*). Phosphorylation at the three sites was not detectable in untreated cells. In the presence of SS-14 phosphorylation at all three sites became detectable within seconds of agonist exposure in both the sst_2_ and the sst_5-2_ receptor. Next, we compared agonist-induced phosphorylation of the wild-type sst_5_ receptor with that of the sst_2-5_ receptor using phosphosite-specific antibodies for pT333 and pT347. Phosphorylation at T347 was already detectable in untreated cells for both receptors (not shown). By contrast, phosphorylation at T333 was not detectable in untreated cells. However, upon addition of SS-14 T333 phosphorylation occurred within a few seconds in both sst_5_ and sst_2-5_ receptors (Figure 1, *right panel*).

**Figure 2:**
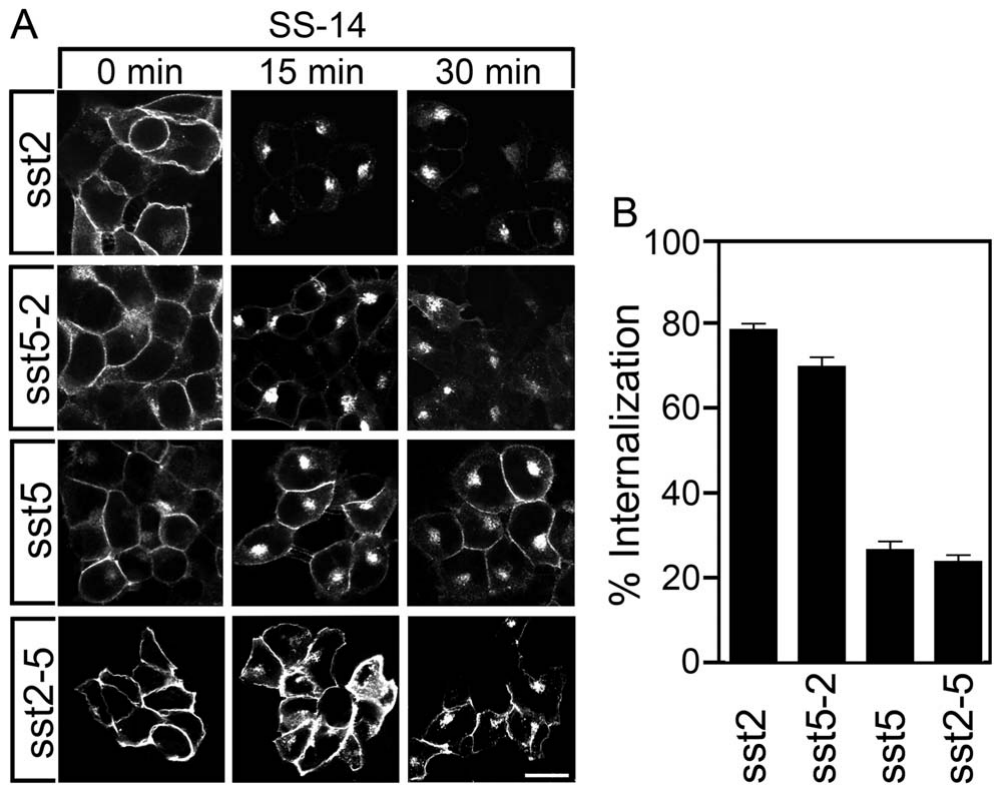
Agonist-induced internalization of sst_2_ and sst_5_ tail-swap mutants. (*A*) Stably transfected HEK293 cells were treated with 1 µM SS-14 for 0, 15 or 30 min. Cells were then fixed, stained with the anti-sst_2_ {UMB-1} or anti-sst_5_ antibody {UMB-4} and examined by confocal microscopy. Shown are representative images from one of at least three independent experiments. *Scale bar*, 20 µm. (*B*) Stably transfected HEK293 cells were treated for 30 min with 1 µM SS-14. Receptor sequestration was measured by ELISA. Data represent per cent internalization of cell-surface receptors in SS-14-treated cells. Data are presented as mean ± SEM from at least three independent experiments performed in quadruplicate.

The sst_2_ receptor and the sst_5_ receptor dramatically differ in the extent of their agonist-induced internalization. In the presence of SS-14, nearly all cell surface sst_2_ receptors are removed from the plasma membrane resulting in a ∼80% loss of surface receptors after 30 min agonist exposure (Figure 2A, B). By contrast, the sst_5_ shows only partial receptor internalization with a large proportion of receptors remaining at the plasma membrane resulting in a maximal internalization of ∼25% after 30 min SS-14 treatment (Figure 2A, B). Interestingly, swapping the cytoplasmic tails completely reversed this trafficking pattern in that the sst_5-2_ receptor revealed nearly complete (∼70%) and the sst_2-5_ receptor partial (∼25%) endocytosis (Figure 2).

**Figure 3:**
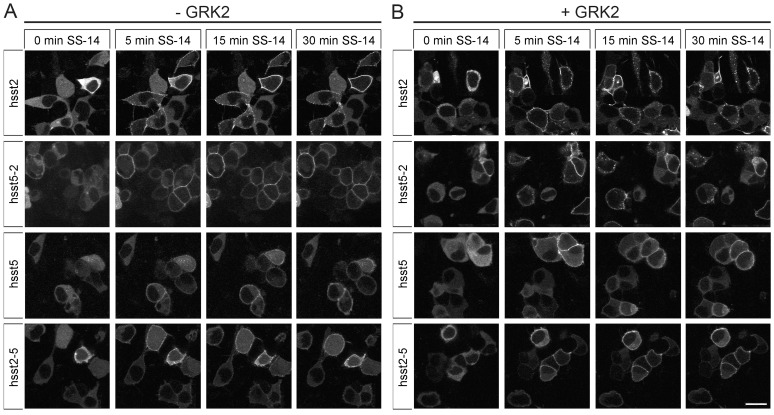
Agonist-induced β-arrestin mobilization of sst_2_ and sst_5_ tail-swap mutants. (*A*) HEK293 cells were transiently transfected with sst_2_, sst_5_, sst_2-5_ or sst_5-2_ and β-arrestin-2-EGFP. The distribution of β-arrestin-2 was visualized sequentially in the same live cells before (0 min) and after the addition of 1 µM SS-14 to the culture medium. Shown are representative images from one of three independent experiments. (*B*) HEK293 cells were transiently cotransfected with sst_2_, sst_5_, sst_2-5_ or sst_5-2_, β-arrestin-2-EGFP and GRK2. The distribution of β-arrestin-2 was visualized sequentially in the same live cells before (0 min) and after (1 to 30 min) the addition of 1 µM SS-14 to the culture medium. Shown are representative images from one of four independent experiments performed in duplicate. *Scale bar*, 20 µm.

We then employed functional β-arrestin-2 conjugated to enhanced green fluorescent protein (EGFP) to visualize the patterns of β-arrestin mobilization in live HEK293 cells. In the absence of agonist, β-arrestin-2-EGFP was uniformly distributed throughout the cytoplasm of the cells (Figure 3). The addition of saturating concentrations of SS-14 (1 µM) to the human sst_2_ receptor induced a rapid redistribution of β-arrestin-2 from the cytoplasm to the plasma membrane resulting in robust fluorescent staining outlining the cell shape (Figure 3A). Overexpression of GRK2 led to the formation of stable complexes between the sst_2_ receptor and β-arrestin-2 that appeared as punctuate staining within the cytoplasm at later time points (Figure 3B). The addition of 1 µM SS-14 to the human sst_5_ receptor induced a redistribution of β-arrestin-2 from the cytoplasm to the plasma membrane that was less pronounced compared to that seen in sst_2_-expressing cells (Figure 3A). Although over- expression of GRK2 clearly facilitated β-arrestin-2 recruitment in sst_5_-expressing cells at early time points, it did not lead to a redistribution of β-arrestin-2-EGFP into the cytosol at later time points (Figure 3B). Again, swapping the cytoplasmic tails led to a complete reversal of the β-arrestin trafficking patterns of these two receptors (Figure 3A, B).

**Figure 4:**
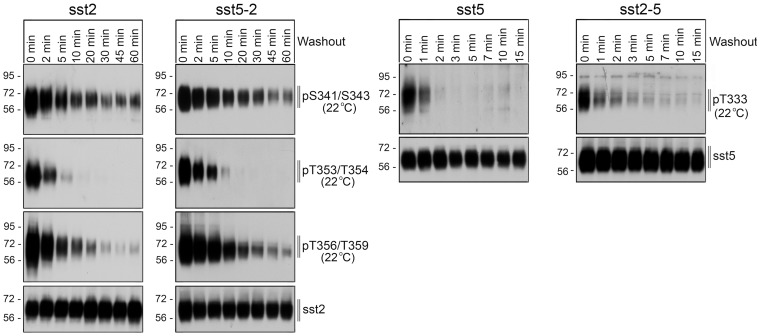
Dephosphorylation of sst_2_ and sst_5_ tail-swap mutants. Stably transfected HEK 293 cells were exposed to 1 µM SS-14 for 5 min, washed and incubated at room temperature in the absence of agonist for the indicated time periods. Cells were lysed and immunoblotted with the indicated phosphosite-specific antibodies. Blots were then stripped and reprobed with the phosphorylation-independent anti-sst_5_ antibody {UMB-4} or anti-sst_2_ antibody {UMB-1} to confirm equal loading of the gels. Shown are representative results from one of three independent experiments. The position of molecular mass markers is indicated on the left (in kDa).

The sst_2_ receptor and the sst_5_ receptor also dramatically differ in their patterns of dephosphorylation and recycling. Interestingly, dephosphorylation of individual sst_2_ phosphate acceptor sites occurs with distinct temporal dynamics. Whereas T353/T354 dephosphorylation occurred rapidly (∼5 min), T356/T359 dephosphorylation was delayed (∼20 min) and S341/S343 dephosphorylation is only observed after extended SS-14 washout (∼60 min) (Figure 4). When sst_5_-expressing cells were exposed to SS-14 for 5 min, washed and then incubated in agonist-free medium, T333 dephosphorylation occurred very rapidly (∼2 min) (Figure 4). In contrast, T347 phosphorylation was although to a lesser extent still detectable even after prolonged incubation in the absence of agonist (not shown). Analysis of chimeric receptors under identical conditions showed that transplantation of the sst_2_ tail to the sst_5_ receptor led to an sst_2_-like dephosphorylation profile (Figure 4). Conversely, transplantation of the sst_5_ tail to the sst_2_ receptor led to an sst_5_-like dephosphorylation profile (Figure 4).

**Figure 5:**
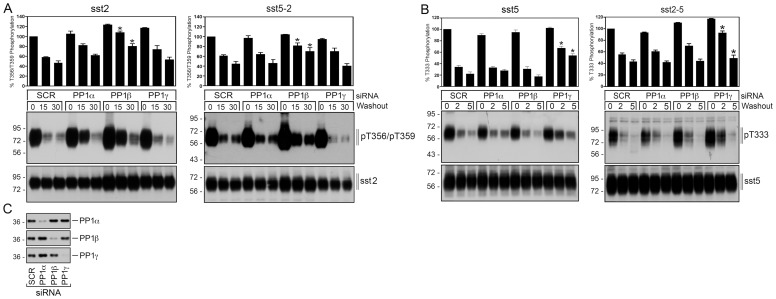
PP1 specificity of sst_2_ and sst_5_ tail-swap mutants. (*A*) HEK 293 cells stably expressing the sst_2_ receptor or the sst_5-2_ tail-swap mutant were transfected with siRNA targeted to PP1α, PP1β, PP1γ or non-silencing siRNA control (SCR) for 72 h and then exposed to 1 µM SS-14 for 5 min. Cells were washed three times and then incubated for 0, 15 or 30 min in the absence of agonist. Cells were lysed and immunoblotted with anti-pT356/T359 antibody {0522}. Blots were stripped and reprobed with the phosphorylation-independent anti-sst2 antibody {UMB-1} to confirm equal loading of the gels. (*B*) HEK 293 cells stably expressing the sst_5_ receptor or the sst_2-5_ tail-swap mutant were transfected with siRNA targeted to PP1α, PP1β, PP1γ or non-silencing siRNA control (SCR) for 72 h and then exposed to 1 µM SS-14 for 5 min. Cells were washed three times and then incubated for 0, 2 or 5 min in the absence of agonist. Cells were lysed and immunoblotted with anti-pT333 {3567} antibody. Blots were stripped and reprobed with the phosphorylation-independent anti-sst5 antibody {UMB-4} to confirm equal loading of the gels. Receptor phosphorylation was quantified and expressed as percentage of maximal phosphorylation in SCR-transfected cells, which was set at 100%. Data correspond to the mean ± SEM from three independent experiments. Results were analyzed by two-way ANOVA. (*C*) siRNA knockdown of PP1 was confirmed by Western blot using isoform-specific PP1 antibodies. The positions of molecular mass markers are indicated on the left (in kDa).

Finally, we examined the PP1 specificity of the sst_2_ receptor and the sst_5_ receptor as well as their respective tail-swap mutants. To date, three distinct catalytic subunits α, β and γ are known for PP1 [13]. To elucidate which of these PP1 isoforms is involved in sst_5_ dephosphorylation, we performed siRNA knockdown experiments. As depicted in Figure 5, PP1β knockdown resulted in a robust inhibition of sst_2_ dephosphorylation. In contrast, transfection of PP1α or PP1β siRNA did not result in a significant inhibition of sst_2_ dephosphorylation (Figure 5). For the sst_5_ receptor only PP1γ knockdown resulted in a detectable inhibition of its dephosphorylation, while transfection of PP1α or PP1β siRNA had no effect (Figure 5). Interestingly, swapping the cytoplasmic tails conferred PP1γ specificity to the sst_2_ receptor and PP1β specificity to the sst_5_ receptor (Figure 5). These results suggest that PP1 specificity of individual somatostatin receptor subtypes is primarily determined by their different carboxyl-terminal receptor domains.

## Discussion

Although the regulation of agonist-induced phosphorylation and internalization has been studied in detail for many GPCRs, the molecular mechanisms and functional consequences of receptor dephosphorylation are far from understood. We have recently observed that closely related somatostatin receptor subtypes can be dephosphorylated by distinct PP1 isoforms. However, it is not known which GPCR domain directs the engagement of specific PP1 isoforms to the receptor. The major finding of this study is that carboxyl-terminal regions of different somatostatin receptor subtypes is a major determinants for their PP1 selectivity. This conclusion is based on the observation that transplantation of the sst_2_ tail to the sst_5_ receptor led to a predominant dephosphorylation by PP1β, whereas transplantation of the sst_5_ tail to the sst_2_ receptor led to a predominant dephosphorylation by PP1γ. Moreover, swapping the cytoplasmic tails led to a complete reversal of the trafficking profiles of these two receptors.

The remarkable selectivity in the recruitment of specific PP1 catalytic subunits to individual somatostatin receptor subtypes is surprising. PP1 catalytic subunits bind to their regulatory subunits and some substrates in a mutually exclusive manner through a conserved RVxF motif. The three isoforms of the PP1 catalytic subunit share greater than 90% sequence identity, including the regions that interact with the RVxF sequence [13]. However, neither the human sst_2_ nor the human sst_5_ receptor has a potential PP1-binding motif in its carboxyl-terminal tail suggesting that somatostatin receptors do not bind to PP1 exclusively by the canonical RVxF motif. Instead, association of PP1 may occur directly through a noncanonical interaction or multiple weak interactions or indirectly via one or more regulatory subunits of PP1. Such targeting PP1 subunits are prime candidates to bring phosphatases in proximity to phosphorylated GPCRs. Nevertheless, the identity of the targeting PP1 subunits remains to be elucidated for both sst_2_ and sst_5_.

Somatostatin receptor subtypes exhibit strikingly different β-arrestin trafficking patterns. The sst_2_ receptor is a prototypical class B receptor that is phosphorylated at clusters of carboxyl-terminal serine and threonine residues. In turn the sst_2_ receptor forms stable β-arrestin complexes, co-internalizes with β-arrestin and recycles slowly. By contrast, sst_5_ is a prototypical class A receptor in that its endocytosis is driven by phosphorylation of a single threonine residue. The sst_5_ receptor then forms unstable ß-arrestin complexes, internalizes without β-arrestin and recycles rapidly. Thus, our finding that swapping the cytoplasmic tails led not only to reversal of the PP1 specificity but also to a reversal of the β-arrestin trafficking profiles of somatostatin receptors suggests a simple model in which fast recycling class A receptors are preferentially dephosphorylated by PP1γ, whereas slow recycling class B receptors are preferentially dephosphorylated by PP1β. So far only few bona fide GPCR phosphatases have been identified [14] [15] [1] [2] [4]. However, it should be noted that this hypothesis is supported by our recent observation that the μ-opioid receptor, which is a prototypical fast recycling class A receptor, is rapidly dephosphorylated by PP1γ [3]. However, it should be noted that PP1α was identified as GPCR phosphatase for the thyrotropin-releasing hormone receptor [4], suggesting that different phosphatases can interact with different GPCRs to mediate their dephosphorylation. It is also possible that distinct phosphatase activities mediate dephosphorylation of plasma membrane receptors versus internalized receptors.

GPCR dephosphorylation has long been viewed as an unregulated process with little or no functional implications. Nevertheless, more recent evidence suggests that PP1ß-mediated dephosphorylation is involved in fine-tuning unconventional ß-arrestin-dependent GPCR signaling. Indeed, inhibition of PP1ß expression results in a specific enhancement of sst_2_-driven ERK activation [1]. Given that arrestin-dependent signaling is initiated by binding to phosphorylated receptors, this finding suggests that PP1ß-mediated GPCR dephosphorylation limits β-arrestin-dependent signaling by disrupting the ß-arrestin-GPCR complex.

In conclusion, different GPCRs can recruit specific PP1 isoforms for their rapid dephosphorylation with remarkable selectivity. This GPCR phosphatase specificity is primarily determined by carboxyl-terminal receptor domains. Recruitment of different GPCR phosphatases has in turn profound consequences for the dephosphorylation dynamics and trafficking patterns of GPCRs.
